# Agreement Between a Subjective Single-Item Socioeconomic Status and Wealth Index in the Addis Health and Demographic Surveillance System (Addis-HDSS), Addis Ababa, Ethiopia

**DOI:** 10.4314/ejhs.v34i2.4S

**Published:** 2024-12

**Authors:** Hanna Gulema, Walelegn Worku Yallew, Nebiyou Fasil, Yoseph Yemane Berhane, Semira Abdlemenan, Hanna Yemane Berhane, Sitota Tsegaye, Dongqing Wang, Uttara Partap, Wafaie Fawzi, Meaza Demissie, Alemayehu Worku, Yemane Berhane

**Affiliations:** 1 Department of Global Health and Health Policy, Addis Continental Institute of Public Health, Addis Ababa, Ethiopia; 2 Department of Epidemiology and Biostatistics, Addis Continental Institute of Public Health, Addis Ababa, Ethiopia; 3 Department of Nutrition and Behavioral Science, Addis Continental Institute of Public Health, Addis Ababa, Ethiopia; 4 Department of Global and Community Health, College of Public Health, George Mason University, Fairfax, Virginia, United States of America; 5 Department of Global Health and Population, Harvard T.H. Chan School of Public Health, Harvard University, Boston, Massachusetts, United States of America

**Keywords:** Wealth index, socioeconomic status, urban, household income adequacy, Addis Ababa

## Abstract

**Background:**

Measuring household economic status is crucial, as it is a key determinant of health. In low-income settings, no single measure of economic status is universally accepted. This study aims to assess the agreement between a single-item tool for measuring socioeconomic status (SES) and the wealth index.

**Methods:**

The Addis Health and Demographic Surveillance System (Addis-HDSS), based in Addis Ababa, Ethiopia, collects data on various socioeconomic indicators, including income, expenditure, and asset ownership. In this study, a single question, “Does your family's income cover basic needs?” was used as a proxy for SES. The percent agreement with the wealth index was calculated, and significance was assessed using the Pearson chi-square test. Scale reliability was evaluated using Cronbach's alpha (α).

**Results:**

Data from 30,533 households showed strong agreement (α = 0.925) between self-reported income adequacy and wealth for both lower and higher wealth groups. The highest agreement was found between the lowest wealth index and “very low” income adequacy (93.84%) and between the highest wealth index and “high” income adequacy (89.47%) (p < 0.001).

**Conclusion:**

The single-item SES measure showed good agreement with the wealth index in an urban setting. This simple tool can effectively identify vulnerable populations for targeted health interventions. Further research is needed to assess its applicability in other contexts.

## Introduction

Socioeconomic status (SES) is a critical determinant of health (1). Assessing economic status at the community level can be challenging due to the limitations of traditional methods, such as income and expenditure data, which are often time-consuming, costly, and prone to inaccuracies due to underreporting or measurement errors (2).

Income and wealth are commonly used measures of SES (3). Income refers to the money a household earns, while wealth is the total value of household assets minus liabilities (4). However, these measures do not account for family size or other social obligations, which are especially important in low-income settings (5). In response, researchers have employed proxy indicators, such as self-reported income adequacy, educational level, occupation, and housing ownership, to assess SES (6). People with higher education, better jobs, and quality housing tend to have better health outcomes (7,8).

While household income is often used to measure SES, it is not always accurately reported (9). The wealth index, which relies on asset ownership, objective measure, and lengthy questionnaires, is often seen as a more reliable SES measure (1,7). However, measuring wealth is time-consuming and costly, and communities may be reluctant to report detailed asset information(2,10). Additionally, in low-income countries, household assets may not accurately reflect true economic status. For example, a mobile phone worth less than $10 may not be comparable to a smartphone valued at $1,000, and assets may lose value over time. Similarly, income, especially from informal sources, is difficult to measure accurately.

Given these challenges, simpler indicators of SES that rely on individuals' judgment of income adequacy may be more feasible and reliable for population-based health survey (11). This study aims to assess the agreement between a simple self-reported indicator of income adequacy and the more traditional wealth index in an urban population.

## Methods

**Study design and population**: The Addis Health and Demographic Surveillance System (Addis-HDSS) conducted a complete housing and population census from December 2022 to January 2023. This longitudinal cohort includes all residents of the Yeka sub-city in Addis Ababa. The census covered 30,533 households, representing over 100,000 individuals. Household heads or adult household members were the primary respondents.

**Data collection and procedures**: Data were collected using a structured electronic questionnaire administered via the Open Data Kit (ODK) application. The questionnaire captured self-reported economic status and sociodemographic information. Data collectors and supervisors received training on survey procedures, ethical considerations, and the use of the questionnaire.

**Economic status measurement**: Two methods were used to assess economic status-

Wealth Index: Principal component analysis (PCA) was used to calculate the wealth index, categorizing individuals into five quintiles based on household assets, such as type of housing, ownership, number of bedrooms, water and sanitation facilities, car ownership, and regular bank savings.

Single-Item Question: Respondents were asked, “Does your regular monthly income cover the basic needs of your family?” with response options: “Not at all,” “Yes, minimally,” “Yes, moderately,” or “Yes, adequately.”

**Data analysis**: Cross-tabulation was used to examine the relationship between the single-item measure and the wealth index. Chi-square tests assessed whether the percent agreement was statistically significant, with significance set at p < 0.05. Cronbach's alpha (α) was calculated to assess scale reliability. Statistical analysis was performed using STATA version 14.

**Ethical considerations**: Ethical approval was granted by the Ethics Review Committee of the Addis Continental Institute of Public Health (ACIPH/IRB/003/2022). Written informed consent was obtained from all participating households.

## Results

A total of 30,533 households were assessed for economic status. The distribution of responses differed between the single-item measure and the wealth index. According to the single-item measure, 7,213 households (23.62%) reported that their income was grossly insufficient to meet basic needs. Another third, 10,196 households (33.39%), indicated that their income barely covered basic needs. The remaining households reported more sufficient incomes: 8,953 (29.32%) reported moderate adequacy, and 4,171 (13.66%) reported that their income adequately met their basic needs. In contrast, the wealth index classified households into nearly equal quintiles, with around 20% in each category.

Demographically, the majority of household heads had a secondary school education (33.52%), followed by those with a college or university education (30.46%) and primary school education (29.12%). The majority of household heads were male (60.67%). The mean household size was 4.38, with a standard deviation of 1.85 (Mean 4.38+SD1.85) (Table 1).

**Table 1:** Economic characteristics of households in the Addis HDSS, Addis Ababa, Ethiopia (n30, 533)

Variables	Response	Frequency (%)
Wealth Index in quintile	Lowest	6,636(21.73)
	Second	5,588(18.30)
	Middle	6,103(19.99)
	Fourth	6,102(19.98)
	Highest	6,104(19.99)
Does your regular monthly income cover the basic needs of the family?	Not at all	7,213(23.62)
	Yes, minimal	10,196(33.39)
	Yes, moderately	8,953(29.32)
	Yes, adequate	4,171(13.66)
Household headship	Male	18,326(60.67)
	Female	11,880(39.33)
Average family size	Mean(±SD) =4.38±1.85	
Educational level of head of the household	Pre-school	189 (0.71)
	Primary school	7,709 (29.12)
	Secondary school	8,875 (33.52)
	Vocational/technical	1,638 (6.19)
	College/University	8,066 (30.46)

**Percent agreement between the single economic assessment and wealth quantites**: The cross-tabulation showed consistent agreement between the wealth index and the reported adequacy of family income to cover basic needs. The chi-square test also revealed a significant association between reported income adequacy and the wealth index (Pearson chi-square, p < 0.001) (Table 2).

**Table 2:** Wealth index and reported family income cover the family's basic needs of the family. Addis Ababa, Ethiopia 2023 (n=30,533)

Variables	Response	Wealth Index				

		Lowest # (%)	Second # (%)	Middle # (%)	Fourth # (%)	Fifth # (%)
Does your regular monthly income cover the basic needs of the family	Not at all	2,804	1,768	1,439	916	286
		(42.25)	(31.64)	(23.58)	(15.01)	(4.69)
	Yes, minimal	2,356	2,200	2,404	2,132	1,104
		(35.50)	(39.37)	(39.39)	(34.94)	(18.09)
	Yes, moderately	1,292	1,332	1,783	2,261	2,285
		(19.47)	(23.84)	(29.22)	(37.05)	(37.43)
	Yes, adequate	184	288	477	793	2,429
		(2.77)	(5.15)	(7.82)	(13.00)	(39.79)

(Pearson chi-square P < 0.001)

**Further analysis of monthly income adequacy and Wealth Index**: Further analysis focused on the categories of low and high-income adequacy, as well as the lowest and highest wealth index categories. A higher percentage agreement of 93.8% was observed between the reported very low-income adequacy and the lowest wealth groups. Similarly, 89.47% agreement was found between high-income adequacy and the highest wealth groups (Cronbach's Alpha, α = 0.925).

## Discussion

This study revealed a significant association between reported family income adequacy and the wealth index. The single self-reported question was particularly effective in differentiating households by economic status, especially in the lowest and highest categories.

The results suggest that simple economic assessments, such as asking about family income adequacy, can provide a cost- and time-efficient way to understand urban economic trends. The single question, relying on families' judgment of income adequacy, performed well in distinguishing households, aligning closely with the wealth index (7,12,13). One reason for this effectiveness may be that households are more comfortable reporting their economic status in broad terms, without providing detailed information. Many households are reluctant to disclose income details due to concerns over taxes or the sources of their income (14).

In contrast, the wealth index treats all assets as having equal value, which is not always the case. Additionally, the wealth index's classifications are context-specific: a household in a high wealth category in one neighborhood may not be considered wealthy in a different area. This limitation, along with the complexity of using the wealth index in large health surveys, which requires asking many questions and the unreliability of responses has been noted in other low-income countries (2). The single-item measure, which agrees well with conventional economic assessments, can be added to health surveys without significant additional cost or time. Furthermore, the results from this simple tool are comparable across different contexts.

This finding underscores the value of simple economic assessments for small-scale studies (4,15). Such assessments allow researchers to gain insights into the economic conditions of a community without the need for extensive or costly data collection (16). Similarly, urban socioeconomic measurements can help identify households in poverty, enabling targeted resource allocation (6,12). With further geo-cluster analysis, it could also be used to identify areas with the highest concentration of impoverished households. Moreover, the simple tool can help evaluate the effectiveness of policies and programs aimed at improving the lives of low-income households (17,18) . It may also play a key role in identifying and addressing health disparities and inequities in healthcare access (9,19,20).

Understanding the socioeconomic composition of urban areas is critical. Socioeconomic disparities often lead to unequal health outcomes and unequal access to healthcare (17,21,22). This study, conducted with a large sample size, provides robust findings. However, there are limitations to using a single question. It may be susceptible to social desirability bias, as some respondents may feel embarrassed to report income inadequacy, especially if they own visible assets. Additionally, “adequacy” is subjective and can vary depending on how households define their basic needs.

In conclusion, the single-item tool, when compared to the more complex wealth index, demonstrated excellent agreement. It effectively identified households in the study area's lowest and highest economic segments. Further research is needed to evaluate the tool in other urban and rural settings.
